# Thirteen year follow up of long term treated psychotic disorder: Personality aspects

**DOI:** 10.1192/j.eurpsy.2021.1369

**Published:** 2021-08-13

**Authors:** T. Fagerberg, J.P. Gustavsson, I. Agartz, E. Jönsson

**Affiliations:** 1 Department Of Clinical Neuroscience, Karolinska Institutet & Stockholm Health Care Services, Stockholm, Sweden; 2 Department Of Clinical Neuroscience, Karolinska Institutet, Stockholm, Sweden

**Keywords:** Swedish universities Scale of Personality (SSP), schizophrénia, Stability of personality traits, Personality

## Abstract

**Introduction:**

Psychotic disorders often cause a drastic change in the life situation of the affected individual. Personality is an aspect that can affect the symptoms and social function in psychotic disorders.

**Objectives:**

No study has examined stability of personality traits exceeding five years in patients with schizophrenia. The aim of this study was to investigate the stability of personality traits over a 13-year period among patients with psychotic disorder and healthy individuals and to evaluate case-control differences.

**Methods:**

At three occasions during a 13-year period patients with psychotic disorders (n=28) and non-psychotic individuals (n=57) completed Swedish universities Scales of Personality (SSP). For all the individuals within- and between-subject analyses were performed at three occasions for all 13 subscales and the three overall factors of SSP. Correlations, means and SDs were calculated.

**Results:**

Tests of within-subject correlations showed differences in two subscales: Lack of Assertiveness, which were influenced by age and Physical Trait Aggression, where patients ratings were stable, whereas controls rated themselves less aggressive at higher age. Between-subjects correlations showed differences regarding any of the parameters diagnosis, time, age, gender or age x gender in nine of the 13 subscales as well as in factor Neuroticism.

**Conclusions:**

Long term follow-up showed a generally high stability of personality traits measured with SSP, especially among patients. Between-subject analyses over the 13 years showed that patients differed compared to controls for the SSP factor Neuroticism as well as the subscale Detachment, which is in accordance with previous studies.

